# Duodenal angiosarcoma can be misdiagnosed as a Dieulafoy’s lesion 

**Published:** 2020

**Authors:** Afshin Amini, Elliot Koury, Zahra Vaezi, Jeffrey Melnick, Andrew Su, Elie Chahla

**Affiliations:** 1 *Department of Medicine, St. Luke’s Hospital, Chesterfield, MO, USA*; 2 *Department of Medicine, Zahedan University of Medical Sciences, Zahedan, Iran*; 3 *Department of Pathology, St. Luke’s Hospital, Chesterfield, MO, USA*; 4 *Division of Gastroenterology and Hepatology, Department of Medicine, St. Luke’s Hospital, Chesterfield, MO, USA*

**Keywords:** Duodenal, Angiosarcoma, Dieulafoy’s lesion

## Abstract

Angiosarcomas are soft-tissue neoplasms that originate from the vascular epithelium. The most commonly involved sites include the skin and subcutaneous tissues. In the GI tract, generally, angiosarcomas involve the spleen and liver, although locations in the small intestine and colon have been very occasionally reported. In the present study we report the unusual case of a man with duodenal epithelioid angiosarcoma, presenting with anemia and recurrent upper gastrointestinal bleeding, which was initially misdiagnosed as a Dieulafoy’s lesion. It is important to consider the diagnosis of gastrointestinal malignancy, including unusual neoplasms such as angiosarcomas, in the setting of anendoscopic appearance such as hemorrhagic nodule, purpuric mass and/or recurrent bleeding lesions that are persistent despite repeat interventions. In such cases, a biopsy should be considered to confirm the diagnosis.

## Introduction

 Angiosarcomas are extremely vascular malignancies, originating from endothelial cells that are categorized as lymphangiosarcomas and hemangiosarcomas, consistent with endothelial histology. They represent around 1-2% of all soft tissue sarcomas and 5% of all malignant skin neoplasms (1). The most commonly involved sites include the skin and subcutaneous tissues. However, due to the pervasiveness of the blood and lymphatic systems, these tumors may arise in any location in the body (2). Angiosarcomas of the gastrointestinal (GI) tract are more uncommon but have previously been described in the literature (3). GI angiosarcomas can be primary or metastatic. However, due to the small number of GI angiosarcoma cases, there is no existing data to represent the common sites of angiosarcoma with a tendency to metastasize in the GI tract (2). In the GI tract, generally, angiosarcomas involve the spleen and liver, while locations in the small intestine and colon have been very occasionally reported (4). Mucosal angiosarcoma can typically be diagnosed by endoscopic mucosal biopsies with pathologic examination, but submucosal lesions may need an endoscopic ultrasound to obtain tissue for pathologic diagnosis (2). 

Here we report the unusual case of a duodenal epithelioid angiosarcoma in a man presenting with anemia and recurrent upper GI bleeding, who was initially misdiagnosed as having a Dieulafoy’s lesion. 

## Case Report

**Figure 1:**
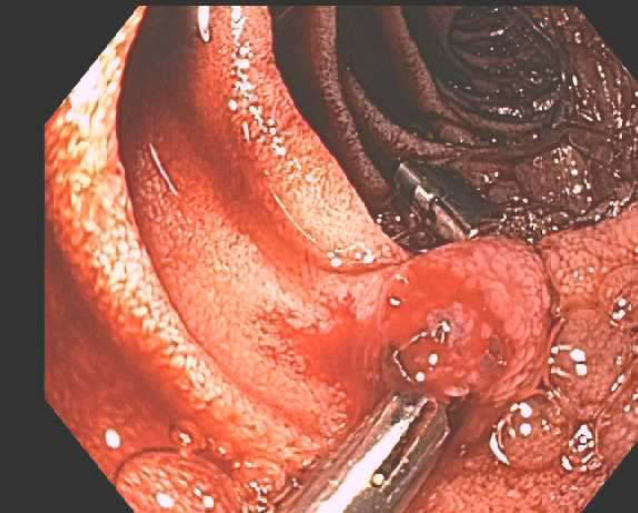
A single 5 mm raised actively bleeding lesion was found in the 2nd part of the duodenum. It already had two clips placed on it. To stop active bleeding, two more hemostatic clips were successfully placed

**Figure 2:**
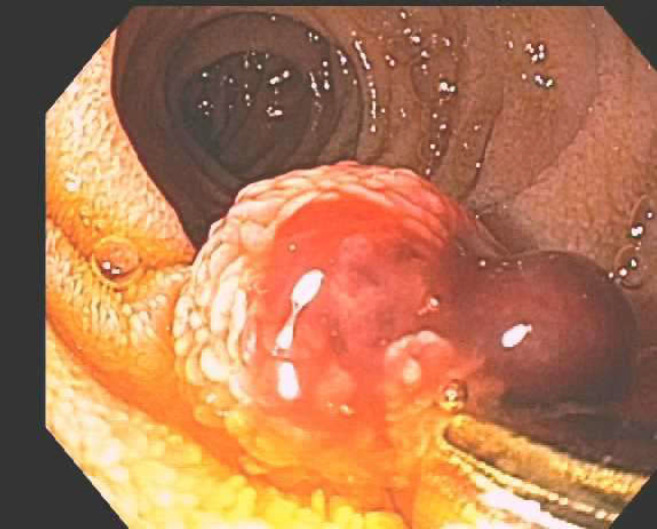
Red blood was found in the second part of the duodenum. For hemostasis, two hemostatic clips were successfully placed

The case is an 82-year-old male patient with a past medical history; significant past illnesses being recurrent upper GI bleeding, a stroke with residual left-sided hemiparesis, hypothyroidism, hyperlipidemia, abdominal aortic aneurysm, and hypertension presenting with melena. Over the past year, the patient had presented several times with melena, and a bleeding lesion was identified in esophagogastroduodenoscopy (EGD) in the second part of the duodenum, which was determined to be a Dieulafoy’s lesion. After the initial diagnosis of Dieulafoy’s lesion, bleeding was controlled with two clips (Figure 1). Recurrent bleeding later prompted repeat EGD and additional clipping of the same lesion (Figure 2).

Further bleeding later that week prompted embolization by interventional radiology. The patient presented this time with melena of 1-week duration. He denied any nausea, vomiting, abdominal pain, NSAID use, and significant alcohol use. He had initially presented to his primary care physician and was found to have a hemoglobin level of 6.9. He was given one unit of packed red blood cells (pRBCs) as an outpatient and was discharged home due to hemodynamic stability. Over the next two days, the patient developed worsening weakness and fatigue with continued melanic stool and presented to the emergency department with a hemoglobin level of 5.6. However, blood pressure and other vitals were stable. He received two units of pRBCs as well as IV pantoprazole in the emergency department and would receive three more throughout the admission. Endoscopy at this time revealed that the previously identified lesion had grown in size from 5 mm to 11 mm. The prior hemoclips were in place, but the lesion was still oozing. An additional clip was placed, and a biopsy was taken (Figue 3). 

**Figure 3:**
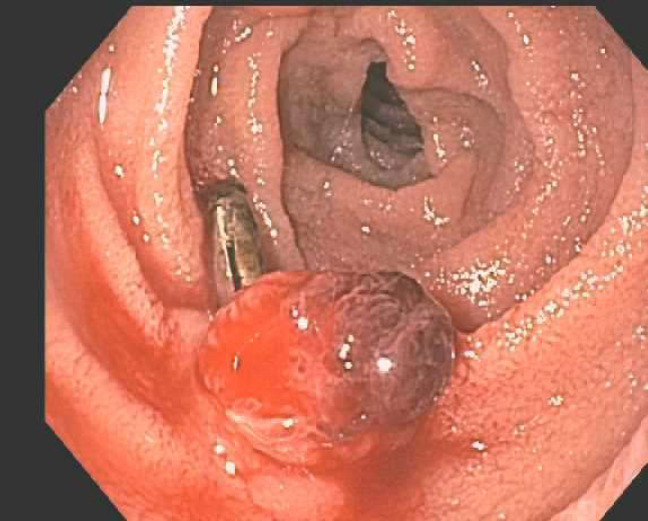
A single 11 mm mucosal nodule with a localized distribution was found in the 2nd part of the duodenum. Area was successfully injected with 2 mL India ink for tattooing. For hemostasis, one hemostatic clip was successfully placed. There was no bleeding during the procedure. Biopsies were taken with cold forceps for histology

**Figure 4:**
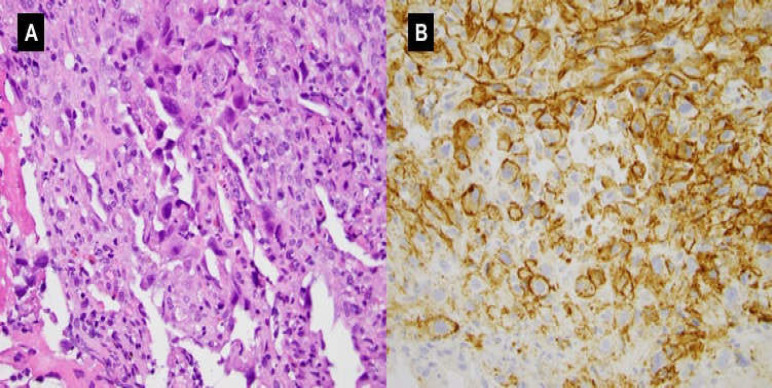
A.H&E at 400x (original magnification) showing area with vasoformative features.B.CD31 immunohistochemical stain at 400x (original magnification) showing membranous and cytoplasmic staining

On histopathology, morphologic and immunophenotypic findings strongly supported the diagnosis of epithelioid angiosarcoma (Figure 4.A, B). The patient elected to seek a second opinion and treatment at an other hospital and was discharged with stable vital signs. He would receive chemotherapy with gemcitabine and taxotere followed by surgical resection with Roux-en-Y procedure and G-tube placement. 

## Discussion

Angiosarcomas are aggressive mesenchymal sarcomas that originate from endothelial cells. These neoplasms, especially the cutaneous type, occur more commonly in the elderly population, in Caucasians, and are three times more likely to affect males than females. They are also known to have a propensity to metastasize and carry a poor prognosis (5). They represent 5% of all malignant skin cancers and 1-2% of all soft tissue sarcomas (1). 

There are four variants of skin angiosarcoma that are recognized. These include scalp and face, lymphedema associated, radiation-induced, and epithelioid angiosarcoma, with the head and neck variant being the most commonly seen. The development of these tumors has been linked to a history of radiation exposure, environmental toxins, foreign bodies, chronic lymphedema, and certain genetic factors (6). Chronic lymphedema, radiodermatitis, and immunosuppression often predispose patients to angiosarcomas in other locations. For example, radiation-induced angiosarcoma most commonly affect the breast. In the setting of lymphedema, the development of angiosarcomas is referred to as Stewart-Treves syndrome. Although the association between lymphedema and angiosarcoma is clear, the mechanism remains unknown (5). 

Due to the pervasive distribution of blood vessels and lymphatics in the body, angiosarcomas can develop in any location. Subtypes include cutaneous, visceral, and soft tissue, with cutaneous and subcutaneous tumors being the most commonly seen. Visceral angiosarcomas, as reported in this case, represent between 15% and 47% of angiosarcomas and are the most difficult to diagnose (6). Visceral angiosarcomas have previously been reported in the breast, heart, lung, liver, spleen, adrenal glands, and ovaries. Angiosarcomas of the gastrointestinal tract, especially of the small bowel, are rarely reported but have been described in scattered case reports (6). These tend to have a very aggressive course characterized by high rates of metastasis and, therefore, poor prognosis. Also, GI angiosarcomas may be found in multiple sites along the GI tract, making it difficult to distinguish between primary and metastatic lesions (7).

The GI lesions can appear in endoscopy as a centrally ulcerated nodule, hemorrhagic nodule, purpuric mass, and submucosal mass.

Gastrointestinal stromal tumors (GIST), GI Kaposi’s lesions, and GI leiomyoma can be misdiagnosed as GI angiosarcoma due to a similar endoscopic appearance and clinical presentation. Endoscopic biopsies with histopathologic examination can distinguish GI angiosarcoma from other similar GI lesions (2). Histologically, angiosarcomas contain numerous vasoformative structures, with immunoreactivity for endothelial cell markers such as CD31 and CD34 (3).

Skin angiosarcomas often present with an insidious onset of violaceous rash, or as nodules or plaques that may bleed or ulcerate in more advanced lesions (8). GI angiosarcomas may present with nonspecific early symptoms, including nausea, vomiting, abdominal pain, gastrointestinal bleeding, anemia, fatigue, and weakness (6). In our case, the patient presented with only the symptom of recurrent GI bleeding that was persistent despite repeated clipping. In terms of treatment, there are no current standard guidelines. When in the early stages, treatment in general consists of surgical resection with the option for adjuvant radiation therapy. The more extensive disease often requires chemotherapy or radiation (8). Despite treatment, recurrence rates range from 72% to 84%. Chemotherapy and other medical treatments have shown varying levels of success. These include docetaxel and/or paclitaxel, propranolol, and targeted biologic therapy such as bevacizumab and imatinib (5). Management of small intestinal angiosarcomas involves controlling bleeding and anemia, followed by resection and/or chemotherapy. Adjuvant radiation therapy may also be employed in multifocal angiosarcomas though this is often avoided in radiation-induced angiosarcomas. Adjuvant chemotherapy regimens are similar to those used in cutaneous angiosarcomas (6).

In conclusion, as demonstrated in this case, it is important to consider the diagnosis of gastrointestinal malignancy, including unusual neoplasms such as angiosarcomas, in the setting of an endoscopic appearance such as hemorrhagic nodules, purpuric mass and/or recurrent bleeding lesions that are persistent despite repeated interventions. In such cases, a biopsy should be considered to confirm the diagnosis.

## Conflict of interests

The authors declare that they have no conflict of interest.
